# Reports of Meetings

**Published:** 1952-04

**Authors:** 


### *Reports of Meetings*

### CLINICAL SOCIETY OF BATH

### The Medical Hazards Confronting the Modern Aviator

Dr. Lewis Scott White discussed *The Medical Hazards Confronting the Modern Aviator*, on December 14th, 1951.

The vast changes which had taken place since the war were perhaps not fully realized. Speed, height and versatility of both civilian and service aircraft had increased to such an extent as to make the strain on pilots correspondingly greater, and the problems of aviation medicine very much more complex.

The main factors were, firstly, rapid changes in pressure due to increased rates of climb and descent and, in pressurized aircraft, the sudden loss of cabin pressure from accident or enemy action. Secondly, changes in motion.

These factors had always been dangerous, but increasing speeds and decreasing safety margins, increased the risk of a serious outcome. In a lateral plane, changes in motion were well withstood by the human body. Downward forces in the vertical plane (i.e. + ve G) were countered with limited success by pressure suits, horizontal posture, etc. Upward forces (i.e. — ve G) were less well tolerated. Whereas the former caused first *greying out* due to loss of colour vision, then *blacking out* and final unconsciousness, recovery usually took place on removal of the stress in good time. On the other hand, with negative G a *red out* occurred, leading to cerebral haemorrhage and death. In preventing these conditions many factors were involved, including good health, general fitness and constant practice.

The difficulties were increased by the use of jet aircraft which, although simpler, quieter and less vulnerable to fuel fires, called for exceptional speed of thought, ability to fly by instrument and ground control, and the strictest flying discipline.

The effect of ultrasonic noise on the ears and higher centres had probably been exaggerated. Usually it arose only in factories where the noise of engines under test was constantly varying through wide frequency ranges.

Cockpit temperature control at heights beyond the protection of the atmosphere involved special problems. Both cooling and heating might be required; a particular difficulty had been the tendency of perspex surfaces and instrument dials to ice-up during descent through cloud. This difficulty was heightened in jet aircraft by the poor forward vision, although vertical and lateral vision were good. Accurate instrument flying was essential, and it was found that specially sensitive instruments had to be developed. The time available for reaction was very small at jet speeds, and instrument flying was therefore very fatiguing to the pilot.

The problem of escape from a damaged fighting jet aircraft was important and it was impossible for the crew to climb from the aircraft without receiving fatal injuries. At present the chief development lay in methods of ejecting the pilot and his seat from the aircraft by a small explosive charge and subsequent rapid free descent to a reasonable atmospheric pressure when a parachute could be used. The force of the ejecting charge had to be most carefully calculated, and compression fractures of the vertebral bodies had occurred in some cases.

Dr. Scott White implied in his final remarks that speeds and stresses were rapidly passing beyond the limitations of the human pilot and that complete mechanical control from the ground is the most probable outcome.

### DEVON AND EXETER MEDICO-CHIRURGICAL SOCIETY

### Tonsils and Adenoids: dogmas and doubts

Mr. Raymond Hinde spoke on *Tonsils and Adenoids: dogmas and doubts*, at a meeting on January 24th, 1952. The physiology of the tonsils and adenoids, he said, is imperfectly understood. The pharyngeal lymphoid tissue is functional only from birth to puberty after which time the adenoids disappear. The lymphoid tissue of Waldeyer's ring differs from that of the lymph nodes in that it has no afferent channels. There is a close morphological relationship between the lymphoid tissue of Waldeyer and that of Peyer's patches; indeed when the tonsils are swollen the Peyer's patches also enlarge.

Tonsillar changes occur at the time of the first dentition at the age of 2½ years: it is suggested that tonsillar enlargement occurring at that time is due to local sepsis in the mouth. If repeated infection by a virulent organism occurs then, permanent pathological changes may follow resulting in the aggregation of masses of lymphoid follicles in a fibrous stroma. But far oftener this tonsillar enlargement is in the nature of a hyperplasia, the sequel of a physiological rather than a pathological change, when it is not possible to tell whether a tonsil is septic merely by looking at it.

A swab from the the tonsils is never sterile. It is normal for a tonsil crypt to contain food debris and organisms; only when a crypt becomes sealed off does it become a source of sepsis. A word of caution must, however, be entered here; the tonsils are a portal of entry for the tubercle bacillus and some 4 per cent. of all tonsils removed show giant cell systems.

Enlarged adenoids need removing only if they obstruct respiration or if they are a source of infection. If the nose cannot be cleared secondary infections such as sinusitis may develop. Before operating it is advisable to wait at least six months so as to be sure that the adenoidal enlargement is really pathological. It is by no means certain that adenoids affect the frequency of colds. On the score merely of size tonsils should be removed only if they meet in the midline or cause dysphagia. Since 1931 there has been a fluctuating decline in the frequency of operation for tonsils and adenoids in the United Kingdom. This decline is due to a more conservative attitude and a more critical selection of cases for operation. If operation is performed it is important that the tonsils and adenoids should be removed completely; remnants left behind provide a greater opportunity for infection than a whole tonsil. It is difficult to decide in individual cases whether the tonsils and adenoids should be left alone or whether tonsils or adenoids only should be removed. At present there are no satisfactory figures on which an accurate judgment can be based.

The influence of environment and home hygiene are considerable. Though it may be difficult or impossible to alter the environment of a family where there are several children, it is possible—and sensible—to treat the one who starts the attacks and leave the others alone.

Mouth breathing may be due to factors other than nasal obstruction and not every child who keeps his mouth open breathes through it. In selecting cases for operation the nose should always be inspected first, then the adenoids using a post-nasal mirror, which is possible in at least 80 per cent. of children.

Before tonsils are removed carious teeth should receive attention. Where operation is considered necessary the optimum time for performing it is when the child has been at school for six or nine months and has had time to develop such immunity as he can to the organisms to which he is exposed. Two further clinical points should be borne in mind: the so called *fixed tonsils* is always one which has been the seat of clinical attacks of infection: otitis media is an indication for the removal of adenoids.

### The Scope of Clinical Electrocardiography

Dr. G. M. Colson discussed *The Scope of Clinical Electrocardiography* at a meeting on February 7th, 1952. Cardiac study, he stressed, demands careful history-taking and complete physical examination. To these, though they provide indispensable supplementary information, electrocardiography and cardioscopy are of secondary importance: but no cardiac study is complete without an electrocardiogram.

In what ways can electrocardiography be of help? Dr. Colson listed the following; by determining the cardiac mechanism; by indicating the presence of myocardial death, ischaemia, or disease of a general nature; by showing the effect of drugs and abnormal electrolytes, or the presence of ventricular strain; by providing evidence of defects in conduction; by establishing in conjunction with other clinical evidence, a diagnosis; and by aiding in the treatment of cardiac disease. It must, however, be borne in mind that electrocardiography cannot establish an aetiological diagnosis, indicate the prognosis or the degree of cardiac compensation, or the presence of valvular lesions.

Thus, a tracing may be normal, slightly or strongly suggestive, or definitely indicative of myocardial disease. Though it may be said that a certain tracing is compatible with a certain disease, for example mitral stenosis, it must be remembered that pulmonary stenosis produces the same picture: the tracing is not therefore diagnostic of either condition. Again, in a case of coronary occlusion, the tracing may be diagnostic of myocardial infarction and will often sharply localize the lesion, but it will give no indication as to whether the underlying pathological process is one of syphilis, arteriosclerosis or periarteritis nodosa. Moreover, in a case of coronary occlusion the electrocardiographic picture may be reverting to normal when the patient may be dying of congestive failure. Thus, a patient may have a normal electrocardiogram and still have gross cardiac disease. On the other hand an abnormal tracing means cardiac disease in the absence of all other positive evidence: Primary pulmonary hypertension gives a tracing identical with that of pulmonary stenosis. Diagnosis and prognosis must therefore be made not by the machine but by the clinician who examines both patient and electrocardiogram.

### What is new in Anaesthesia

Dr. E. J. Powell posed the rhetorical question, *What is new in Anaesthesia?* at a meeting on February 21, 1952. Deep muscular relaxation, he said, could be attained by the use of inhalation anaesthetics but the depth of narcosis entailed was often detrimental to the patient. A fresh approach to the problem had produced the various methods of regional and spinal, sometimes combined with light general, anaesthesia. First results of the use of curare in anaesthesia were reported by Griffith of Montreal in 1942; in this country by Halton and Gray in 1946 who stressed the importance of maintaining adequate respiratory exchange when using $\beta$ tubocurarine. The value of this new method having been recognized many substitutes have since been clinically assessed. Such are decamenthonium iodide, syncurine, C.10, and gallamine triethiodide. Tubocurine limits the action of acetylocholine upon the end-plate; decamethonium iodide causes depolarization of such a degree that it spreads beyond the end-plate to the adjacent muscle which thus becomes unresponsive to further stimuli—an action analogous to that of an excess of acetylocholine. A new relaxant, succinyl choline, has been reported from Sweden and also from Rome. Its pharmacological action in some ways resembles that of decamethonium iodide but differs in being rapidly hydrolysed at the neuro-muscular junction, probably by cholinesterase. Being transient in its action succinyl choline is of especial value in procedures requiring short periods of deep muscular relaxation.

### The Problems of Geriatrics

Dr. M. C. Binnie spoke of geriatrics, its scope and problems, on March 6th, 1952. Students and nurses in training are scantily taught about the care and management of the elderly sick. The degenerative conditions of old age offer an unlimited field for research as yet hardly explored, despite the certain prospect of an increase in our elderly population. It is vital to our economy to ensure that as many elderly persons as possible should be fit enough to contribute their quota of work beyond the usual age of retirement. Geriatrics should be regarded more as an outlook than as a specialty. Ideally, no elderly sick person should be admitted to a long-term hospital without adequate screening in the general wards of a hospital or elsewhere. A complete assessment of the patient is the keystone of sound geriatrics. This cannot be done at a single clinical examination but may take several weeks. The outstanding contribution so far made by geriatrics is its insistence on early activity. Contractures, bed-sores, incontinence, pulmonary emboli and aspiration pneumonia are some of the sequelae of too prolonged rest in bed. Cases of coronary thrombosis or congestive failure in the aged should be confined to bed for about half the time required by a younger person. Even before he gets up much can be done for the elderly patient by simple physiotherapy. All who deal with him should be encouraged to keep this principle of mobility constantly in mind.

Next in importance comes rehabilitation of body and mind. Occupational therapy especially of a diversional type can do much towards this end. Behind the geriatric unit there must be a good background of social service. The activities of the multitude of bodies and committees engaged in this sphere are at present all too often inco-ordinated. To help the general practitioner with his geriatric work there is need of domiciliary physiotherapists, occupational therapists, and of more nurses trained in geriatric methods. Bluestone in New York has introduced a comprehensive geriatric domiciliary service based on a general hospital, avoiding the needless use of hospital beds both for diagnosis and treatment and thereby saving three-quarters of the cost. Arteriosclerosis is perhaps the gravest clinical problem of geriatrics. Though much can be done for cases of cerebral, coronary and peripheral vascular thrombosis, we are at present unable to prevent relapse or extension just as the patient has reached an ambulant stage. In domiciliary work anticoagulant therapy is not very practicable.

Senile dementia is another pressing problem. Many of these cases do not require certification but may be undesirable or even intolerable in general wards. Degenerative arthritis, chronic respiratory disorders, vertigo and urinary incontinence present great difficulties but none needs to be regarded as hopeless; many of them can be improved, or even cured, by patience and perseverance.

### SOUTH WEST ORTHOPAEDIC CLUB

A meeting of the South West Orthopaedic Club was held at Gloucester on Saturday, November 24th, 1951. Thirty-six members, a record number, attended. Mr. J. Bourdillon was in the chair.

Mr. B. Kyle (Bristol) reported on the results of forty-six tendon grafts for severed flexor tendons of the fingers which had been carried out at Bristol since 1946. The graft had been done not less than six weeks after the original injury. This was necessary in order to allow the tissues to settle down. He recorded a brief outline of the technique. The graft was obtained from the proximal remnant of the sublimis tendon or from the palmaris longus if it was present. The latter was removed with an adequate covering of paratenon. The proximal end of the graft was sutured with a buried wire suture of Bunnell type. No movement was allowed for two weeks. Nerves were repaired at the same time as the tendons. If tendons in two fingers were damaged, only one finger was grafted at one operation.

In sixty per cent a good result was obtained. The commonest cause of failure was end to end suture of tendons of unequal size with resultant adhesion. There is no danger in delaying operation for some time as retraction of the profundus is prevented by the attachment of the lumbrical muscles. One case had been operated on ten years after injury. The sublimis tendon appeared to be superior to the palmaris longus.

Mr. Mervyn Evans (Swansea) advised operative reduction and screw fixation for the supracondylar fracture of the lower end of the humerus. He said that the treatment by activity—the so called " bag of bones " technique, was useful in the patient over forty but the result was imperfect. Operative reduction had failed previously on account of the position of the incision and the amount of trauma resulting from the reduction and the plate and screws inserted. He suggested that the Y should be converted into a simple supracondylar fracture by means of an incision on the medial side of the joint and the insertion of a transverse screw to maintain the reduction of the distal fragments. The supracondylar fracture was then manipulated into good position. A pressure bandage was applied for three weeks and then activity was commenced. He had carried out this operation in six cases. He did not advise it in patients over forty. He showed X-rays and clinical photographs of these cases which had excellent range of movement and perfect anatomical reduction.

Mr. J. Bastow (Bath) discussed the use of the Schanz osteotomy for the late unreduced congenital dislocation of the hip or when a dislocation recurs and a shelf operation is not possible. He claimed that the result would last the patient for a lifetime and it was therefore better than arthroplasty. There was no late arthritis. He said that the completely dislocated hip does not develop arthritis but osteoarthritis of the spine occurs as a result of the lordosis. The osteotomy corrected the latter. The disadvantages were (1) limitation of movement, (2) if done too early the osteotomy angle tends to become reduced and finally, (3) that it prevented a possible arthroplasty operation from being performed later.

Mr. A. O. Parker (Cardiff) believed that in the failed case of congenital dislocation of the hip nothing should be done until growth had been completed. He believed that relief of the lordosis was important and favoured a posterior cuneiform osteotomy to correct this. Miss MAUD FORRESTER-BROWN liked the Lorenz osteotomy better than the Schanz because the latter caused a loss of length.

Miss FORRESTER-BROWN then gave a brief, but entertaining account of her recent flying visit to South Africa. She was most impressed by the kindness she had received everywhere and by the progress which was being made in orthopaedics. She mentioned the research which was going on into virus of poliomyelitis at Johannesburg. She had visited a hospital school at Kimberley which was coping with the disabled, especially spastics. She said that there was still a grave shortage of beds for tuberculosis. The physiotherapy services were being expanded and she was interested to see native warriors who had been trained to become highly efficient masseurs in the course of a few years.

Mr. DILLWYN EVANS (Cardiff) presented the case histories and X-rays of four cases of spinal disease simulating tuberculosis which stimulated much discussion. Three of these subsequently were proved staphylococcal infections. It was generally agreed that a negative Mantoux Test overruled a diagnosis of tuberculosis.

At the clinical session cases were presented. Mr. J. STALLMAN (Gloucester) demonstrated some excellent results of McMurray's osteotomy for osteoarthritis of the hip and convinced many members that cup arthroplasty is not the only operation to be considered in such cases.

Mr. BOURDILLON showed ten cases of slipped upper femoral epiphyses, eight of which had been seen during the last three years. Six cases had open operation and nailing. These results were excellent. Mr. Henry showed several interesting cases, among them a central fracture of acetabulum reduced by open operation and screw fixation with a perfect anatomical and radiological result; also a subluxating carpal scaphoid.

Mr. R. K. DEBENHAM (Birmingham) showed cases of chondro-osteodystrophy, arachnodactyly and diaphyseal aclasis.

After tea a visit was paid to the Gloucester Aircraft Works where the Gloster Meteor Jet Aircraft were seen in all stages of construction.

Later the Annual Dinner of the Club concluded a full but entertaining day of great interest.

### SECOND PATRICK WATSON-WILLIAMS MEMORIAL LECTURE

### Defence of the Air Passages

Mr. V. E. NEGUS delivered the second Patrick Watson-Williams Memorial Lecture on October 18th in Bristol University. In introducing the speaker Professor R. MILNES WALKER recalled that the Negus bronchoscope and oesophagoscope proved how broadly Mr. Negus had interpreted his specialty of ear nose and throat surgery.

Mr. Negus said that students were taught about the nose, sinuses, and the chest in different hospital departments. These structures were all parts of the same system—the air passages.

There were lessons to be drawn from animals. Thus the climbing perch, which had both gills and a respiratory diverticulum capable of taking up oxygen from the air, could breathe air so long as the epithelial lining was moist, a principle which held good throughout the animal kingdom. The sense of smell although lying in the air passages played no part in protecting the lungs. Comparative anatomy also showed that the larynx was not primarily a vocal organ but a valve to keep food out of the lungs.

In man it was of paramount importance that inspired air should be warm and moist. If this process failed then the exchange of gases in the lungs would be hampered, cilia which swept away bacteria and debris would cease to function and lyzozyme activity would stop. This air conditioning was a function of the nose and was much affected by the climate. The nose could give up as much as one litre a day. Air could be dry either when it was cold as on Mount Everest or when it was hot as in the Sahara; in either case much water had to be provided by the nose. In moist atmospheres less strain fell on the nasal mucosa. Breathing over-moist air carried a risk of blocking bronchioles by condensation of water vapour.

Mr. Negus then showed his film on " The Action of Cilia ". This film had been made during his work at the Royal College of Surgeons and illustrated beautifully how ciliated epithelium kept the air passages clear. Lamp black in a frog's Eustachian tube was swept away and swallowed. Under high power magnification of small pieces of mucosa from a rabbit's trachea the movements of individual cilia could easily be seen. The film showed most vividly how ciliated epithelium could be hampered by altering the fluid in which it was bathed. If there was too much fluid cilia could no longer sweep it away. They merely churned it up, while in cocaine and irritating solutions cilia were profoundly inhibited.

Mr. Negus pointed out that treatment of diseases of the air passages must be based on physiological principles. Atropine for example could do harm by hindering the action of cilia. Cilia likewise could not complete their work if there was a gap in their ranks from granulation tissue or large operations. Again extensive cauterizing was illogical.

Mr. Negus finally considered the importance of all this knowledge in civilized society. Central heating without adequate humidification made the air warm but dry. The need to moisten this air was becoming increasingly recognized, particularly by architects and engineers in America.

